# Cellular splicing factor UAP56 stimulates trimeric NP formation for assembly of functional influenza viral ribonucleoprotein complexes

**DOI:** 10.1038/s41598-017-13784-4

**Published:** 2017-10-25

**Authors:** Yifan Hu, Vishal Gor, Kazuya Morikawa, Kyosuke Nagata, Atsushi Kawaguchi

**Affiliations:** 1 Department of Infection Biology, Graduate School of Comprehensive Human Sciences, University of Tsukuba, 1-1-1 Tennodai, Tsukuba, 305-8575 Japan; 2 Department of Infection Biology, Faculty of Medicine, University of Tsukuba, 1-1-1 Tennodai, Tsukuba, 305-8575 Japan; 3 Transborder Medical Research Center, University of Tsukuba, 1-1-1 Tennodai, Tsukuba, 305-8575 Japan

## Abstract

The influenza virus RNA genome exists as a ribonucleoprotein (RNP) complex by interacting with NP, one of virus-encoded RNA binding proteins. It is proposed that trimeric NP is a functional form, but it is not clear how trimeric NP is formed and transferred to RNA. UAP56, a cellular splicing factor, functions as a molecular chaperone for NP and is required for the replication-coupled RNP formation of newly synthesized viral genome, but the details of NP transfer to viral RNA by UAP56 is unclear. Here we found that UAP56 is complexed with trimeric NP, but not monomeric NP. Gel filtration analysis and atomic force microscopy analysis indicated that the complex consists of two trimeric NP connected by UAP56. We also found that UAP56 stimulates trimeric NP formation from monomeric NP even at physiological salt concentrations. Thus, UAP56 facilitates the transfer of NP to viral RNA since trimeric NP has higher RNA binding activity than monomeric NP. Further, UAP56 represses the binding of excess amount of NP to RNA possibly by transferring trimeric NP. Collectively, we propose that UAP56 stimulates viral RNP formation through promotion of the assembly of trimeric NP and is important for the structural integrity of NP-RNA complex.

## Introduction

The genome of influenza type A viruses is single-stranded RNAs of negative polarity. The viral genome (vRNA) exists as ribonucleoprotein (designated vRNP) complexes with heterotrimeric viral RNA-dependent RNA polymerases and nucleoprotein (NP)^1^. NP is one of the basic viral proteins and binds single-stranded RNA without sequence specificity. NP is essential to maintain the RNA template in an ordered conformation suitable for viral RNA syntheses^2–6^. Cryo-electron microscopy analysis revealed that the oligomerization of NP is important to form a double helical structure with anti-parallel strand of vRNP^7,8^. Oligomeric NP shows higher RNA binding activity than monomeric NP^9^. Although crystal structures of RNA-free NP showed that NP forms oligomers in the crystalline state, a broad size distribution was observed by gel filtration chromatography in solution at physiological salt concentrations^9–12^, suggesting that NP exists in an equilibrium between monomers and oligomers including trimers and tetramers^13^. Thus, an exact form of NP to be assembled into vRNP remains unknown.

For efficient viral transcription and replication, not only viral factors but also host factors are required. It has been reported that RAF-2p48/NPI-5/UAP56, Tat-SF1, and Prp18 function as molecular chaperones for NP to recruit NP to the viral RNAs^4,14,15^. NP chaperones are also required to suppress the aggregation of NP. UAP56 is a cellular splicing factor belonging to the DExD-box family of ATP-dependent RNA helicase^16^. It is reported that the newly synthesized viral genome is co-replicationally assembled into RNP complex by UAP56^3^. UAP56 binds to NP free of RNA but not NP-RNA complexes^15^. It is also reported that UAP56 interacts with N-terminal region of NP, and NP interacts with C-terminal region of UAP56^15^. However, the molecular mechanism of the transfer of NP to RNA by UAP56 is still unknown.

Here we examined the binding stoichiometry of NP-UAP56 complex using recombinant UAP56 and RNA-free NP. We found that UAP56 stably interacts with trimeric NP, and stimulates the assembly of trimers from monomeric NP. Gel filtration experiments showed that the molecular size of UAP56-NP complex is more than 440 kDa, suggesting that the complex is composed of 6 molecules of NP and 2 molecules of UAP56. By atomic force microscopy (AFM) analysis of NP-UAP56, we found a dumbbell-shaped complex which is considered as two trimeric NP connected by UAP56. We also revealed that UAP56 facilitates NP-RNA complex formation without excessively oligomerized NP-RNA complexes. Taken together, these results suggest that UAP56 stimulates the RNP formation through the assembly of trimeric NP and controls the amount of NP on RNA for the structural integrity of NP-RNA complex for efficient viral RNA synthesis.

## Results and Discussion

### Binding stoichiometry of NP and UAP56 complex

**Figure 1:**
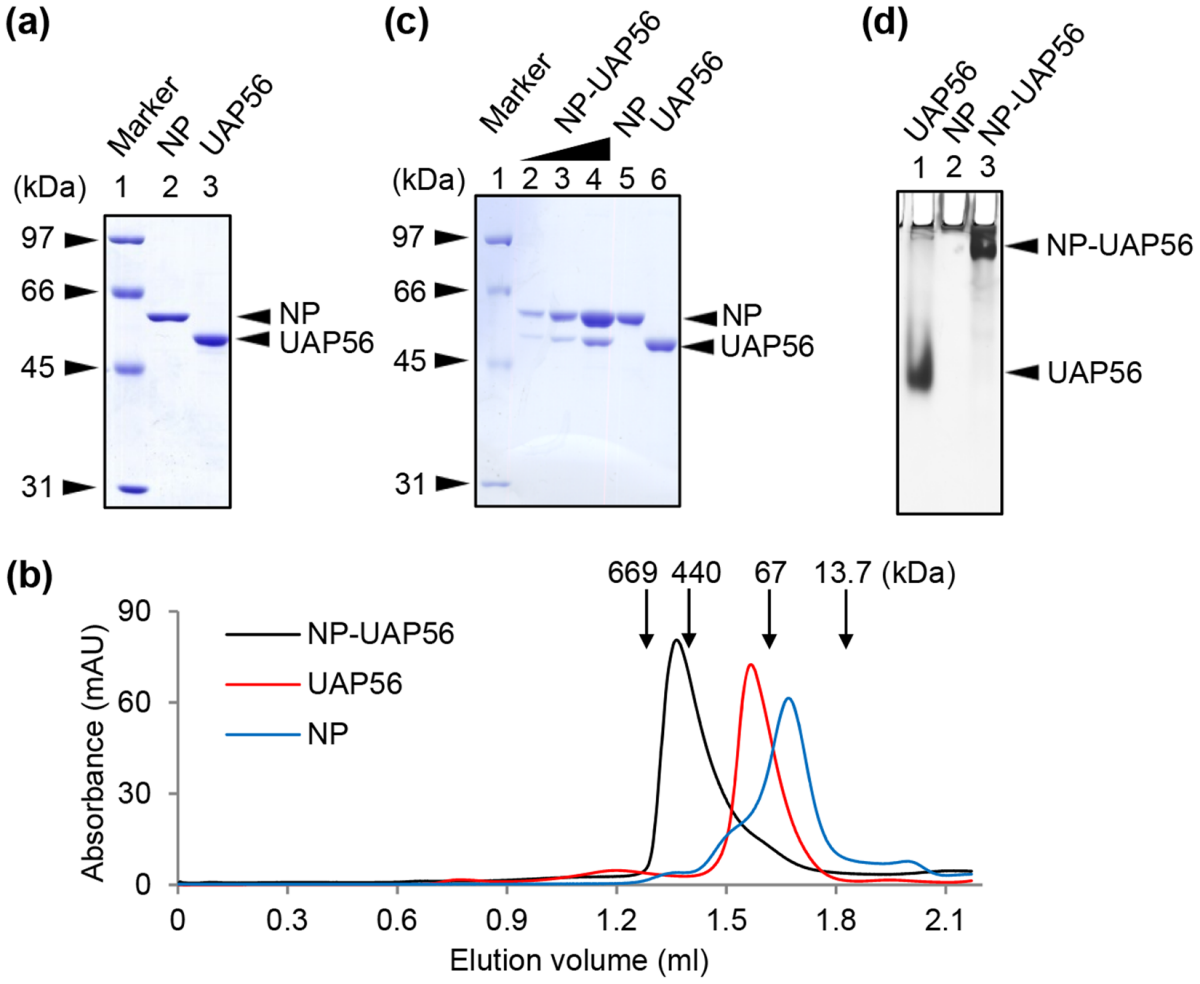
Binding stoichiometry of NP-UAP56 complex. (**a**) SDS-PAGE analysis. The detail of purification scheme is described in Methods. Lane 1, molecular size marker; Lane 2, NP-His; Lane 3, UAP56. (**b**) Gel filtration chromatography. NP-UAP56 complex was partially purified using Sephacryl S-200 gel filtration column. Then, the purified NP-UAP56 complex (black), UAP56 (red), and NP (blue) were eluted from a Superose 6 gel filtration column. The peak positions of protein standards are marked by arrows. (**c**) NP (300 ng), UAP56 (300 ng), and NP-UAP56 complex (66, 200, and 600 ng) were separated on 10% SDS-PAGE and visualized by CBB staining. (**d**) UAP56, NP, and NP-UAP56 complex were analyzed by 6% native PAGE in 0.5 × TBE buffer and visualized by silver staining.

NP-His and GST-UAP56 were expressed in *E*. *coli* BL21(DE3) strain and purified by Ni-NTA resin and GST resin, respectively (Fig. 1a). The GST tag of UAP56 was digested with Prescission protease (Fig. 1a, lane 3). After buffer exchange to a buffer containing 25 mM HEPES-NaOH (pH 7.0), 150 mM NaCl, 1 mM DTT, and 5% glycerol, purified NP-His and UAP56 were incubated at 4 °C overnight. The NP-UAP56 complex was partially purified using Sephacryl S-200 gel filtration column, and then the NP-UAP56 complex was analyzed by Superose 6 gel filtration column (Fig. 1b). In the absence of NP, UAP56 was eluted as a homogenous single peak at elution volume of 1.6 ml, which corresponds to the molecular weight of about 100 kDa. Because theoretical molecular weight of UAP56 is 49.8 kDa, this result suggested that UAP56 forms a dimer. The elution peak of NP free of UAP56 was found at around 63 kDa, suggesting that NP mainly exists as a monomer (theoretical molecular weight is 57.9 kDa). We found that the NP-UAP56 complex was recovered as a single peak at elution volume of 1.4 ml, which corresponds to the molecular weight of more than 440 kDa. CBB staining of the peak fraction showed that the complex consists of NP and UAP56 at a 3:1 molar ratio, indicating that UAP56 interacts with trimeric NP (Fig. 1c, lanes 2–4). Similar results were obtained by an alternative staining method, called VisPRO™ 5 Minutes Protein Stain Kit, which stains the gel but not the protein sample (Supplementary Figure S1). Since the observed molecular weight of NP-UAP56 complex on the gel filtration column was around 440 kDa, it is likely that the NP-UAP56 complex is composed of 6 molecules of NP and 2 molecules of UAP56 with the theoretical molecular weight of 447 kDa. To confirm the binding of NP to UAP56, aliquots of the peak fraction were applied to a 6% native PAGE (Fig. 1d). UAP56 is an acidic protein with isoelectric point of about 5.4 and can be migrated to the cathode side in native PAGE (Fig. 1d, lane 1), whereas NP is hardly migrated to the gel because NP is a highly basic protein with isoelectric point of about 9.6 (Fig. 1d, lane 2). The NP-UAP56 complex showed a slower migration pattern than UAP56 free of NP, and there is no UAP56 free of NP (Fig. 1d, lane 3). These results suggest that UAP56 stably interacts with NP.

### UAP56 binds to trimeric NP but not monomeric NP

**Figure 2:**
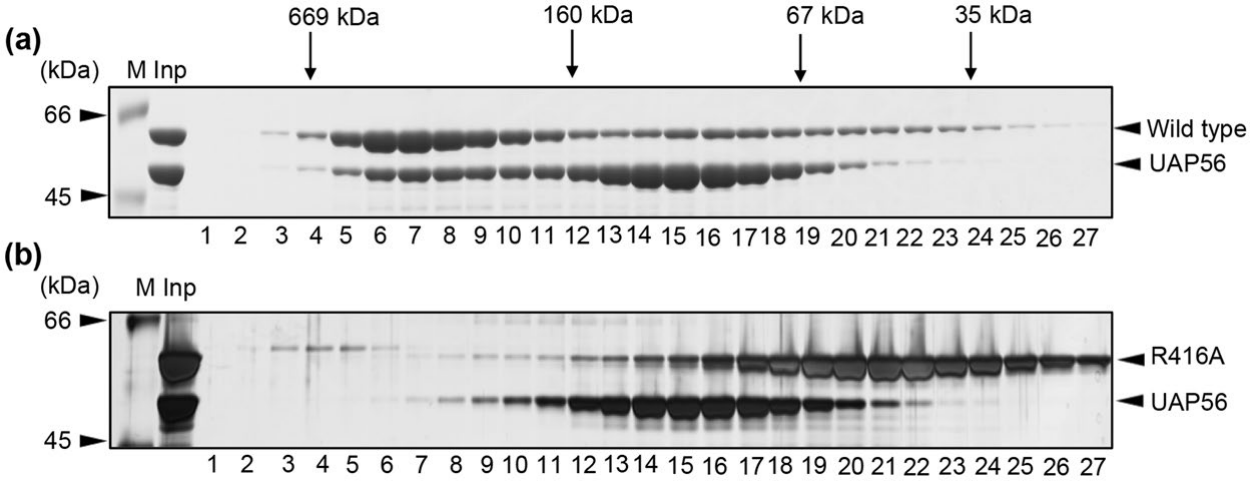
UAP56 interacts with trimeric NP but not monomeric NP. Wild type NP (panel A) and R416A NP mutant (panel B) were incubated with UAP56, respectively. Each sample was subjected to gel filtration chromatography, and the eluted proteins were separated on 10% SDS-PAGE and visualized by CBB staining. The peak positions of protein standards are marked by arrows.

NP consists of head and body domains with a long tail loop, formed by residues 402–428, at the outlying part of RNA-binding groove. The interaction of the tail loop from one NP molecule with a neighboring subunit is important to form homo-oligomers. Next, to examine whether monomeric or oligomeric NP binds to UAP56, we purified a tail loop mutant, R416A, which is a monomeric NP mutant^9,12,17^. UAP56 was incubated with either wild type or R416A mutant at 4 °C overnight. The mixtures were fractionated through chromatography on a gel filtration column, and the fractions were analyzed by 10% SDS-PAGE (Fig. 2). We found that UAP56 was recovered with wt NP in the fractions around 440 kDa (Fig. 2a), but R416A mutant did not (Fig. 2b). This suggests that UAP56 specifically interacts with trimeric NP. It is noted that UAP56 recognizes the N-terminal 188 amino acid residues of NP^15^, suggesting that the tail loop is not the direct binding site for UAP56. It is reported that monomeric NP binds to RNA, and the association of additional monomers to the pre-existing monomeric NP-RNA complex leads to higher order oligomers^9^. This stepwise assembly of NP-RNA complex is thought to be a slow process^9^. In contrast, pre-formed trimeric NP interacts rapidly with RNA^9^. Although our purified NP mainly contains monomeric NP (Fig. 1b), a large part of NP interacting with UAP56 was recovered as trimeric NP as shown in Fig. 1b (Fig. 2a, lanes 6–8). Thus, it is quite likely that UAP56 promotes the trimer formation of NP, and thereby UAP56 facilitates the transfer of NP to RNA as a molecular chaperone.

**Figure 3:**
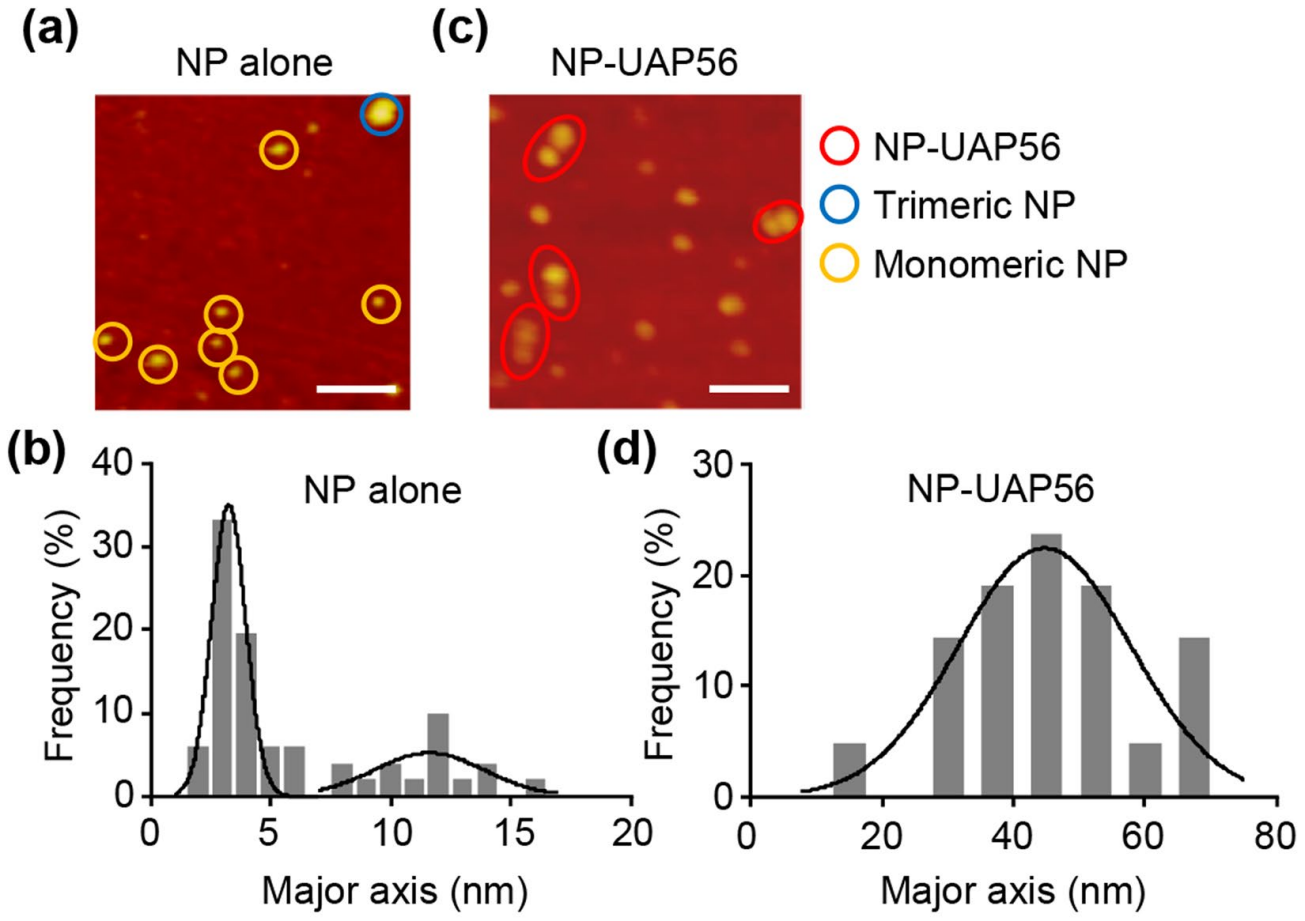
NP-UAP56 was observed to be a dumbbell-shaped complex by AFM. NP alone (panels a and b) and NP-UAP56 complex (panels C and D) were subjected to AFM analysis, respectively. Monomeric NP, trimeric NP, and NP-UAP56 particles are indicated by yellow, blue, and red circles, respectively (panels A and C). Scale bar, 100 nm. The size distribution of NP or NP-UAP56 particles in major axis is shown in panels B and D. Solid lines represent a Gaussian fit.

Next, to demonstrate the protein composition of NP-UAP56 complex, we carried out AFM analyses (Fig. 3). In the absence of UAP56, NP was observed as globular proteins which are approximately either 3–4 nm or 12–13 nm in length (Fig. 3a and b). According to the crystal structure of NP^12^, the size of monomeric NP and trimeric NP is thought to be approximately 5 nm and 15 nm, respectively. This suggests that the smaller particles are monomeric NP, while the bigger ones are trimeric NP. In contrast, NP-UAP56 complex was found as a dumbbell-shaped complex of approximately 40–50 nm in major axis (Fig. 3c and d). Since the size of trimeric NP and UAP56 dimer are is approximately 15 nm and 6 nm^18^ respectively, suggesting that the dumbbell-shaped complex seems to be two trimeric NP connected by UAP56 dimer.

### UAP56-mediated NP-RNA complex formation

**Figure 4:**
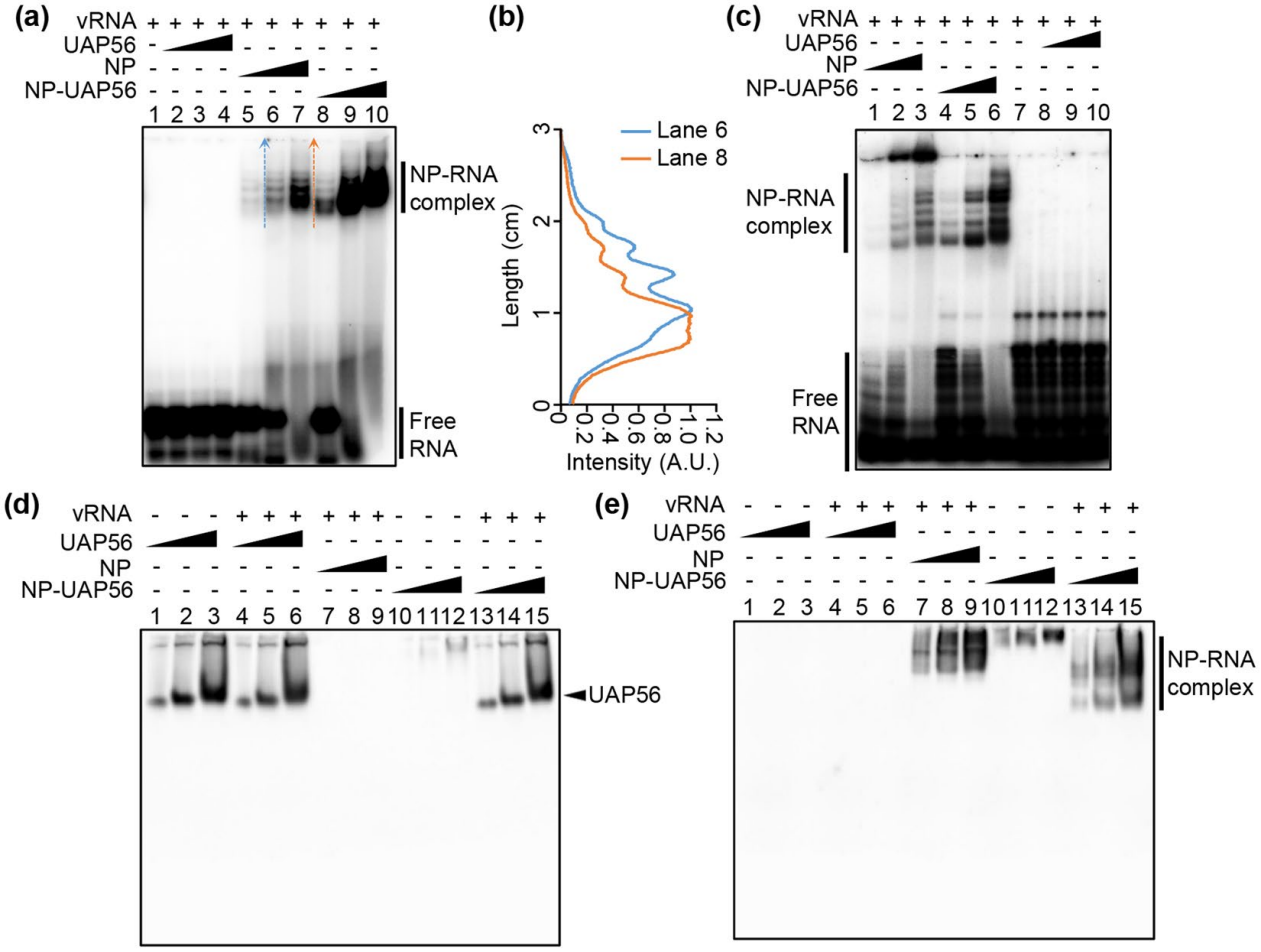
UAP56 mediates the NP-RNA complex formation as a molecular chaperone. (**a**) 0.5 pmol of^32^ P-labeled 53 nt-long model vRNA was incubated with increasing amounts (0.8 pmol, lanes 2, 5, and 8; 2.4 pmol, lanes 3, 6, and 9; 7.2 pmol, lanes 4, 7, and 10) of UAP56 (lanes 2–4), NP (lanes 5–7) or NP-UAP56 complex (lanes 8–10) at 30 °C for 30 min. The samples were analyzed by 6% native PAGE in 0.5 × TBE buffer and visualized by autoradiography. (**b**) The signal intensities of lanes 6 and 8 in panel A were measured by ImageJ software along the dashed arrows shown within the panel A. (**c**) 0.5 pmol of ^32^P-labeled 165 nt-long model vRNA was incubated with increasing amounts (0.8 pmol, lanes 1, 4, and 8; 2.4 pmol, lanes 2, 5, and 9; 7.2 pmol, lanes 3, 6, and 10) of UAP56 (lanes 8–10), NP (lanes 1–3) or NP-UAP56 complex (lanes 4–6) at 30 °C for 30 min. The samples were analyzed by 6% native PAGE in 0.5 × TBE buffer and visualized by autoradiography. (**d** and **e**) NP (0.6 pmol, 1.8 pmol, and 5.4 pmol), UAP56 (0.6 pmol, 1.8 pmol, and 5.4 pmol) or NP-UAP56 complex (0.6 pmol, 1.8 pmol, and 5.4 pmol) was incubated with 0.5 pmol of 53 nt-long vRNA. UAP56 (0.6 pmol, 1.8 pmol, and 5.4 pmol), or NP-UAP56 (0.6 pmol, 1.8 pmol, and 5.4 pmol) was also incubated without 53 nt-long vRNA. The samples were separated on 6% native PAGE in 0.5 × TBE buffer, and the gels were subjected to western blotting using anti-UAP56 (panel D) and anti-His-tag (panel E) antibodies, respectively.

To examine the chaperone activity of the purified NP-UAP56 complex, we performed EMSA with [^32^P]-labeled 53 nt-long (Fig. 4a and b) and 165 nt-long (Fig. 4c) model vRNAs, respectively. The formation of NP-RNA complex was stimulated in the presence of UAP56 (Fig. 4a, compare lanes 5–7 with lanes 8–10; Fig. 4c, compare lanes 1–3 with lanes 4–6). Further, the migration rate of NP-RNA complex in the presence of UAP56 was faster than that without UAP56 (Fig. 4a, compare lane 6 with lane 8; Fig. 4b). In the case of 165 nt-long model vRNA, excessively oligomerized NP-RNA complexes that cannot enter native PAGE were formed in the absence of UAP56 (Fig. 4c, lanes 1–3), whereas the NP-RNA complex assembled in the presence of UAP56 successfully migrated to the gel (Fig. 4c, lanes 4–6). It has been proposed that NP binds to a replicating RNA, and then additional NP molecules are subsequently recruited by the NP-NP oligomerization for the efficient encapsidation of nascent chains^5,17^. Thus, it is possible that UAP56 modulates the self-oligomerization of NP on viral RNAs to assemble the functional RNP complexes by recruiting trimeric NP to viral RNAs. To examine whether UAP56 interacts with NP-RNA complex or not, we carried out the western blotting using antibodies against UAP56 (Fig. 4d) and His-tag (Fig. 4e) after separating the NP-RNA complex by EMSA. UAP56 free of NP was incubated in the absence or presence of 53 nt-long model vRNA, and subjected to EMSA (Fig. 4d, lanes 1–6). The migration rate of UAP56 was not changed in the presence of RNA, suggesting that the negatively charged UAP56 does not bind to RNA as shown in Fig. 4a (lanes 2–4) and Fig. 4c (lanes 8–10). Further, by the addition of RNA to NP-UAP56 complex, UAP56 was not found in NP-RNA complex (compare Fig. 4d, lanes 13–15 with Fig. 4e, lanes 13–15) and migrated to the same position as UAP56 free of NP (compare lanes 1–3 with lanes 13–15 in Fig. 4d). This result suggests that NP-UAP56 complex was dissociated by the addition of RNA as previously proposed^15^.

**Figure 5:**
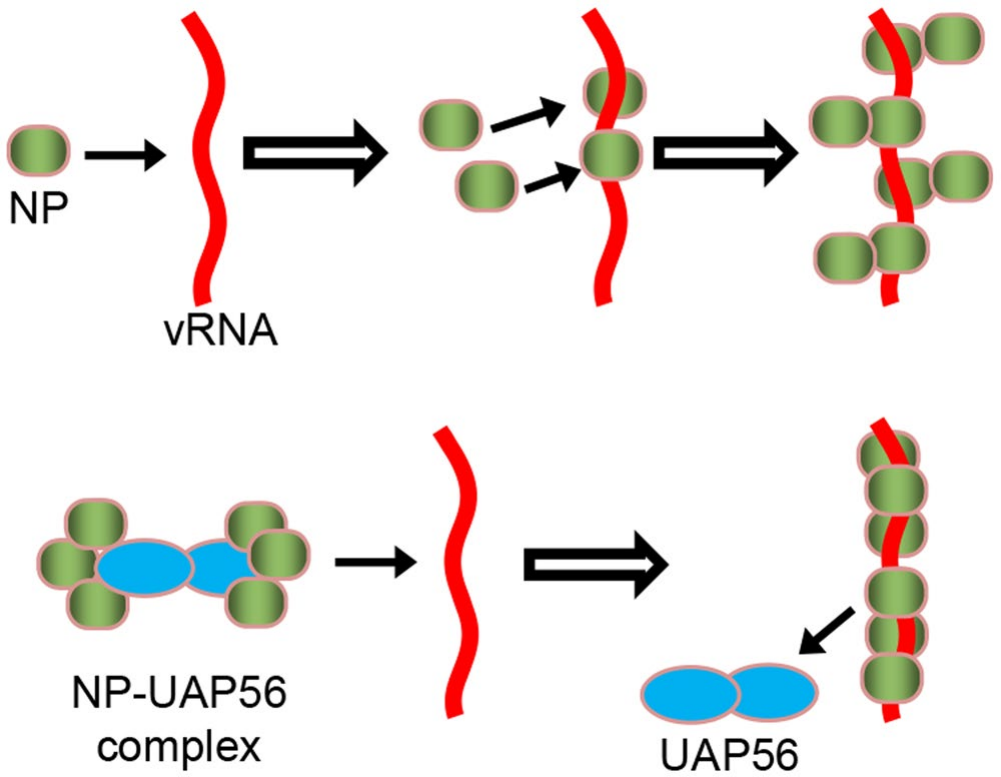
A proposed model. In the absence of UAP56, monomeric NP first binds to RNA, and the additional monomers randomly bind to the monomeric NP on NP-RNA complex, slowly. The dimerized UAP56 proteins bind to trimeric NP, respectively. UAP56 stimulates the transfer of NP to viral RNA by facilitating trimeric NP formation for the structural integrity of NP-RNA complex competent for viral RNA synthesis.

In conclusion, UAP56 forms dimer in solution, and the binding stoichiometry of NP-UAP56 complex is a 1:3 molar ratio, suggesting that UAP56 interacts with trimeric NP. Thus, it is likely that dimerized UAP56 proteins bind to trimeric NP, respectively. We also found that UAP56 stimulates trimeric NP formation from monomeric NP even at physiological salt concentrations. Therefore, UAP56 facilitates the transfer of NP to viral RNA as a molecular chaperone since trimeric NP has higher RNA binding activity than monomeric NP^9^. In the absence of UAP56, monomeric NP first binds to RNA, and the additional monomers randomly bind to the monomeric NP on NP-RNA complex, slowly^9^ (Fig. 5). In contrast, we found that UAP56 represses the binding of an excess amount of NP to RNA possibly by transferring trimeric NP (Fig. 5). This is in good agreement with previous reports that UAP56 provides functional NP-RNA complex competent for viral RNA synthesis^3,15^. Taken altogether, we propose that UAP56 simulates NP-RNA complex formation by recruiting trimeric NP and suppresses the formation of excessively oligomerized NP-RNA complex as shown in the latter model (Fig. 5). Our findings reveal the molecular mechanism of molecular chaperone-dependent ribonucleoprotein complex formation for viral RNA synthesis.

## Methods

### Biological materials

Rabbit anti-UAP56 antibody was prepared as previously described^15^. Mouse anti-His antibody was purchased (Nacalai tesque). Plasmids expressing His-tagged NP and UAP56 were constructed using pET14b and pGEX-2T as previously described^3,15^. For the construction of a plasmid expressing R416A NP mutant, cDNA containing R416A point mutation was amplified from pET14b-NP by overlap PCR with primers 5ʹ-TCAGTACAGGCAAATCTCCCT-3ʹ, 5ʹ-AGGGAGATTTGCCTGTACTGA-3ʹ, 5ʹ-GGGAATTCCATATGGCGTCTCAAGGCACCA-3ʹ, and 5ʹ-CCGGTCGACTAGTGATGATGATGATGATGGCCATTGTCGTACTCCTCTGCATTGTCTCCG-3ʹ. The cDNA was cloned into pET14b at *Nco* I and *Nde* I sites.

### Preparation of recombinant proteins

Plasmids expressing NP-His, R416A NP-His, and GST-UAP56, were transformed into BL21(DE3) strain (Stratagene), respectively. The proteins were expressed in LB medium for 16 h at 18 °C after induction with 0.2 mM IPTG. For NP-His and R416A NP-His, the harvested cells were suspended in lysis buffer A (25 mM HEPES-NaOH [pH 7.9], 500 mM NaCl, 30 mM imidazole, and 5% glycerol) containing 0.1% Triton X-100 and 1 mM PMSF. After lysis by sonication, the lysates were centrifuged at 20,000 × g for 30 min. The supernatants were filtrated with a 0.45-μm filter and subjected to His-tag purification using Ni-NTA agarose beads (GE healthcare). After washing with five column volumes of buffer A, NP proteins were eluted in buffer A containing 300 mM imidazole. To remove the bacterial RNA bound to NP, the NP lysates were treated with RNase A before purification and washed with buffer A containing 1.5 M NaCl during purification. Purified NP with a ratio of the absorbance at 260 nm to that at 280 nm of between 0.57 and 0.59 was used for this study. For GST-UAP56, the cell pellet was resuspended in lysis buffer B (25 mM HEPES-NaOH [pH 7.3], 500 mM NaCl, 1 mM DTT, and 5% glycerol) containing 0.1% Triton X-100 and 1 mM PMSF. After sonication, the lysates were centrifuged at 20,000 × g for 30 min. The supernatants were filtrated with a 0.45-μm filter and subjected to GST-tag purification using glutathione Sepharose beads (GE healthcare). After washing with 5 column volumes of buffer B, GST-UAP56 immobilized on the resin was digested with an appropriate amount of PreScission protease for 12 h at 4 °C. UAP56 without GST tag was collected in the flow through fraction.

### Gel filtration analysis

After buffer exchange of purified NP and UAP56 to buffer C (25 mM HEPES-NaOH [pH 7.0], 150 mM NaCl, 1 mM DTT, and 5% glycerol) using a desalting column (HiPreP 26/10 Desalting, GE Healthcare), purified NP and UAP56 were mixed at a 1:1 molar ratio, and were incubated at 4 °C for overnight. The NP-UAP56 complex was purified by a gel filtration chromatography using the HiPrep 16/60 Sephacryl S-200 HR column (GE Healthcare) on AKTA system (GE Healthcare) or Superose 6 PC 3.2/30 column on SMART system (Amersham Pharmacia) in buffer C. The SDS-PAGE gel of NP-UAP56 was visualized by either CBB staining or VisPRO™ 5 Minutes Protein Stain Kit (Visual Protein). The band intensities were measured by ImageJ software.

### AFM analysis

NP alone and NP-UAP56 complex were subjected to AFM analysis in air at room temperature (Bruker Nanoscope VIII, Bruker). The system was operated in tapping mode with a 100-μm scanner. Probes made of a single silicon crystal with a cantilever length of 129 µm and spring constant of 33–62 N/m (OMCL-AC160TS-W2, Olympus) were used for imaging. Data were collected in the height mode. Images were captured in 512 × 512 pixels and the captured images were flattened and plane-fitted before analysis. The size of the particles was calculated with a correction for tip effect as previously reported^19^.

### Electrophoretic mobility shift assay (EMSA)

Increasing amounts (0.8 pmol, 2.4 pmol, and 7.2 pmol) of NP or NP-UAP56 complex were incubated with 0.5 pmol of^32^P-labeled 53 nt-long model viral genome (5′-AGUAGAAACAAGGGUGUUUUUUCAUAUCAUUUAAACUUCACCCUGCUUUUGCU-3′) or 165 nt-long model viral genome (5′- AGUAGAAACAAGGUCGUUUUUAAACUAUUCGACACUAAUUGAUGGCCAUCCGAAUUUAUGGUCCACGGUGGUUUUUGUGAGUAUCUCGCGGGUGCGAGACUGCGACAUUAGAUUUCUUAGUUCUUUUAUUCUUUCCAUAUUGAAUAUAAUUGACCUGCUUUCGCU-3′) in a buffer containing 50 mM HEPES-NaOH (pH 8.0), 100 mM NaCl, 5 mM MgCl_2_, 1 mM DTT, and 5% glycerol at 30 °C for 30 min. The samples were applied to a 6% native PAGE in 0.5 × TBE buffer and visualized by autoradiography.

### Western blotting assay

UAP56 (0.6 pmol, 1.8 pmol, and 5.4 pmol) or NP-UAP56 complex (0.6 pmol, 1.8 pmol, and 5.4 pmol) was incubated with 0.5 pmol of 53 nt-long model vRNA in a buffer containing 50 mM HEPES-NaOH (pH 8.0), 100 mM NaCl, 5 mM MgCl_2_, 1 mM DTT, and 5% glycerol. The samples were separated on 6% native PAGE in 0.5 × TBE buffer, and the gels were subjected to western blotting using anti-UAP56 and anti-His-tag antibodies, respectively.

## Electronic supplementary material

Supplementary Figure
