# Supplementary material for: Cellular splicing factor UAP56 stimulates trimeric NP formation for assembly of functional influenza viral ribonucleoprotein complexes
Source: Sci Rep. 2017 Oct 25;7:14053. doi: 10.1038/s41598-017-13784-4 (PMC5656576; doi:10.1038/s41598-017-13784-4)
Supplement: Supplementary file 1 — Supplementary Figure [file 41598_2017_13784_MOESM1_ESM.pdf]

Title: Cellular splicing factor UAP56 stimulates trimeric NP formation for assembly of functional influenza viral ribonucleoprotein complexes

Authors: Yifan Hu[1], Vishal Gor[1], Kazuya Morikawa[1,2], Kyosuke Nagata[2*], and Atsushi Kawaguchi[1,2,3*]

Address:

[1]Department of Infection Biology, Graduate School of Comprehensive Human Sciences, University of Tsukuba, 1-1-1 Tennodai, Tsukuba 305-8575, Japan

[2]Department of Infection Biology, Faculty of Medicine, University of Tsukuba, 1-1-1 Tennodai, Tsukuba 305-8575, Japan

[3]Transborder Medical Research Center, University of Tsukuba, 1-1-1 Tennodai, Tsukuba 305-8575, Japan

[*]Correspondence should be addressed to A. K. (e-mail: ats-kawaguchi@md.tsukuba.ac.jp) and K. N. (e-mail: knagata@md.tsukuba.ac.jp)

Contact information:

Atsushi Kawaguchi, Department of Infection Biology, Faculty of Medicine, University of Tsukuba, 1-1-1 Tennodai, Tsukuba 305-8575, Japan Phone: (Japan +81) 29-853-3233, Fax (Japan +81) 29-853-3233, Email: ats-kawaguchi@md.tsukuba.ac.jp

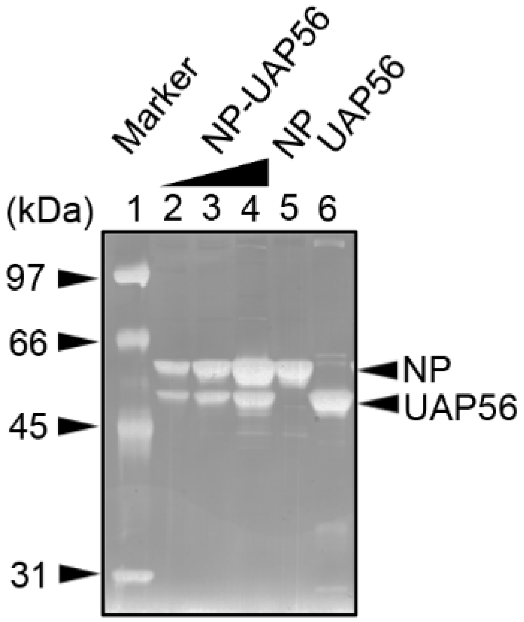

Supplementary Figure S1.

NP (300 ng), UAP56 (300 ng), and NP-UAP56 complex (66, 200, and 600 ng) were separated on 10% SDS-PAGE and visualized by VisPRO™ 5 Minutes Protein Stain Kit.
